# Cholera and Its Prevention

**Published:** 1884-08

**Authors:** 


### Cholera and its Prevention.

The march of cholera in Europe, from the extreme East along the lines of commerce and of travel, is a warning that its progress may not be intercepted by the Atlantic unless vigorous measures are adopted. The experience of Southern Europe seems to expose the fallaciousness of quarantine as a means of prevention of the march of this terrible scourge. The English system, adopted after careful scientific investigations, of even inquisitional inspection of shipping and the personal examination of travelers and their effects, has thus far excluded the disease from England. The subject is one of great importance to health authorities. The port-offices of the large shipping centers on the Atlantic, as well as the Pacific, are entrusted with a grave responsibility, and only the strictest vigilance will guard our shores from an invasion. In view of the interest which the public as well as the profession naturally take in this matter, we have taken occasion to examine into the measures recommended for adoption by health authorities, and beg to present our readers a digest of such as we think will be of practical use. We regret that the National Board of Health have failed to take notice of this important matter, and to issue directions for the guidance of local health boards. The Michigan State Board of Health, which, under the direction of Prof. Kedzie, is always in the vanguard on sanitary matters, have issued a valuable circular containing ample rules and valuable suggestions, which we place before our readers:

"The investigations by Dr. Koch show that the bacillus of cholera can live and reproduce its kind indefinitely in certain, but not in all substances outside the body, namely, in certain alkaline but not in acid solutions; and as the normal condition of the stomach is acid, that it cannot live in the human stomach in its normal condition. The intestinal juices being normally alkaline, the bacillus can, probably, reproduce itself therein without limit whenever it can pass through the stomach. This makes it of especial importance that in times of danger from cholera, the stomach should be kept in its naturally good condition.

" Because of the possibility that the cholera bacillus may find lodgment and multiply in various kinds of moist filth, it is important that everything about the house, cellars, barns, premises, alleys and streets, should be cleaned up and kept dry, and as clean as possible, and that there should be a general disinfection of all places liable to become infected. Especially should privy-vaults, sewers, cess-pools, drains, and similar places be thoroughly and often disinfected with a strong solution of copperas, which may be made acid by the addition of sulphuric acid. The cholera bacilli are said to thrive in nutritive alkaline solutions, and the contents of most privy-vaults are alkaline; hence, the importance of such thorough and frequent disinfection as shall kill any one of the germs which may find lodgment there·

RESTRICTION OF CHOLERA.

" One of the chief means of restricting cholera is to disinfec immediately and thoroughly all the discharges from those sick with cholera, or with the premonitory diarrhœa, and to disinfect or burn at once completely all their cast-off clothing, bedding, etc.

" The fecal discharges are not as infectious when first voided as they soon become, hence the importance of immediate disinfection. Thrown, without disinfection, into a privy-vault, cess-pool, or sewer, the fluids vomited, and especially the discharges from the bowels of a cholera patient, may soon infect all its contents, and render it a source of infection to those who approach.

" All the discharges from the body—the vomit, the discharges from the bowels, etc., should be received into vessels containing some concentrated disinfectant, such as chloride of zinc, copperas, or sulphate of zinc, to which may be added sulphuric or other mineral acid.

"Clothing soiled by a cholera patient, if laid aside and allowed to remain moist, soon becomes especially dangerous. It is, therefore, important that all such articles be immediately burned or placed in a strong disinfecting solution until such time as they can be burned, or boiled, washed and dried. (Dr. Koch's experiments indicate that the bacilli of cholera are destroyed by being thoroughly dried for three hours or more.)

"The diarrhœa preceding cholera is frequently painless, and there is, therefore, during the occurrence of cholera, great danger of cholera being spread by the discharges of persons yet able to travel about. During the first stages of cholera, and especially during the initiatory diarrhœa, prompt medical treatment is important and useful, both for the benefit of the individual and as a means of checking the spread of the disease.

"It has been a practice in England, and should be the practice everywhere, when a man is found sick with cholera, to learn by inquiry what privies he has visited, and at once send an officer on the back track to disinfect them. For reasons just stated, notice should at once be sent to the board of health of a locality from which a case of cholera has come.

"Great care should be had to prevent the contamination of the water-supply by choleraic discharges, as by drainage into wells, springs, or other water-supply, from a privy-vault, sewer-drain, or cemetery. The use of water from a source liable to be infected with cholera excreta should be promptly stopped.

"Bodies of those dead from cholera should be wrapped in a cloth wet with a zinc solution, and at once buried; the zinc solution to be made in proportions as follows: Water, one gallon; sulphate of zinc, eight ounces; common salt, four ounces.

DISINFECTION OF CLOTHING, ROOMS, ETC.

"It is best to burn all articles which have been soiled by a person sick with cholera. In the glowing fire of a large furnace is a good place to burn clothing. Great care should be taken to burn quickly and thoroughly whatever is burned, and not simply warm up and spread the infection.

"Articles too valuable to be destroyed should be exposed for one hour to a dry heat of from 240° F. to 250° F., or three hours at a temperature of 150° F., or be treated as follows:

"Cotton, linen, flannels, blankets, etc., should be treated with the boiling-hot zinc solution (one-half of the strength of that mentioned in the preceding paragraph), introducing them piece by piece, securing thorough wetting and boiling for at least half an hour. Heavy woolen clothing, silks, stuffed bed-covers, beds, and other articles which cannot be treated with the zinc solution, should be hung in the room during fumigation, pockets being turned inside out, and the whole garment being thoroughly exposed. Afterward they should be hung in the open air, beaten, and shaken. Carpets are best fumigated on the floor, but should afterward be removed to the open air and thoroughly beaten. In no case should the thorough disinfection of clothing, bedding, etc., be omitted.

"After a death or recovery from cholera, the room in which there has been a case of cholera, whether fatal or not, should, with all its contents, be thoroughly disinfected by exposure for several hours to strong fumes of burning sulphur, and then it should for several hours, if possible for days, be exposed to currents of fresh air.

"Because of the innumerable ways in which the infection may be scattered about the house and premises where there has been a case of cholera, the entire house and out-buildings, including cellar, wood-shed and privy, may well be disinfected.

"Rooms to be disinfected must be vacated. For a room about ten feet square, at least two pounds of sulphur should be used; for larger rooms, proportionately increased quantities, at the rate of two pounds for each one thousand cubic feet of air-space.

"Close the rooms as tight as possible, place the sulphur in iron pans which will not leak, supported upon bricks, or over a sheet of zinc, set the sulphur on fire by hot coals or with the aid of a spoonful of alcohol lighted by a match, be careful not to breathe the fumes of the burning sulphur, and when certain the sulphur is burning well, leave the room, close the door, and allow the room to be closed for twenty-four hours.

"Privies, cess-pools, drains, water-closets, sewers, gutters, etc., should be frequently and liberally treated with copperas solution made in the proportion of one and one-half pounds of copperas to one gallon of water."

The King's and Queen's College of Physicians of Ireland have issued the following suggestions for the use of the public, as to the treatment of early or suspicious symptoms at seasons when cholera is threatened, or is epidemic:

"The College advise no alteration in the habits of living, where these have previously been moderate and regular. All excess should be carefully avoided, especially in the use of alcoholic drinks, as it is, of noted experience, the intemperate who most certainly fall victims to the most fatal type of cholera, as of other epidemic diseases.

"All food likely to cause indigestion or bowel complaint should be carefully avoided, particularly fruit in a large quantity or in an unripe, decayed, or unsound state; likewise, fish or meat when in the least tainted.

"Water should be used for drinking purposes only after being boiled; and, in consequence of the possibility of milk being diluted with infective water, it likewise should be boiled before use.

"Strict personal cleanliness should be practiced, and the clothing should be adapted to the season and weather.

"All debilitating causes should be carefully avoided, such as excessive and long-continued fatigue and fasting, overcrowding, exposure to moist, stagnant air, or to air loaded with organic effluvia.

"During the prevalence of cholera, any person affected by any of the following complaints should at once obtain medical advice: 1. Diarrhœa, or looseness of the bowels. 2. Vomiting or sickness of stomach. 3. Pains in the stomach or bowels. 4. Pains or cramps in the legs. While such aid is being obtained, the patient should be put to bed immediately, and warmth should be encouraged by the application of heat to the body and limbs. Also, in case of sickness of stomach, a large mustard poultice should be applied over the abdomen. In the event of cramps ensuing, diligent rubbing of the limbs should be resorted to.

"Hot brandy punch should be administered in small and repeated doses, and the diet should be restricted to rice, milk flavored with cinnamon and brandy, or arrow-root prepared with milk or port wine.

"Should relief not be shortly obtained, and the looseness of the bowels continue, ten grains of aromatic chalk-powder with opium, or twenty drops of dilute sulphuric acid, with five drops of laudanum, should be administered in a tablespoonful of water, to which a little brandy may be added. This dose, which is intended for an adult, may be repeated every hour for three doses.

"The discharges from the bowels should be disinfected, and disposed of as quickly as possible. A wineglassful of the following disinfectant mixture should be poured into the vessels used by the patient, namely: Common sulphate of iron (green copperas), one ounce; carbolic acid, a quarter of an ounce; water, twenty fluid ounces, or one imperial pint.

"Infected bedding, and all articles of clothing worn by the sick, should be destroyed by fire."

